# University College Hospital

**Published:** 1840-01-01

**Authors:** 


### UNIVERSITY COLLEGE HOSPITAL.

### DEATH FROM THE USE OF THE TWISTED SUTURE FOR A VARICOSE VEIN.*

A dyer, aged 59, of intemperate habits, was admitted June 19, 1839, with an ulcer on the right leg, and many tortuous veins connected with it. The sore was at first covered with the water dressing, and after the man's bowels had been cleared out with a dose of calomel and senna mixture it was determined to try to cure the varicose veins with twisted sutures, and three were at once made; one about four inches above the knee, and two others below it.

Much suffering and swelling followed the suture in the thigh; it was withdrawn on the 26th.

27th. Very severe pain in the left shoulder and arm. Two or three shivering fits, each of half an hour's duration, succeeded by heat and other febrile disturbance.

28th. Shoulder and arm still exceedingly painful. Another shivering fit this morning. Considerable redness and hardness at inner side of the thigh, in the course of the great saphænal vein. The two other pins were removed. On the 8th of July, the man was attacked with diarrhœa. There was low fever. On the 12th, abscesses in the thigh in the situation where the upper suture had been applied. Openings made for the discharge of the matter. On the 17th, he died.

*Dissection.*—Surface generally of a yellow tinge. *Thorax:* lungs extensively adherent to the parietes of the chest, especially the left, and the adhesions not of long standing. Tubercular deposits in various stages within the lungs. In the pericardium three ounces of serous fluid. Heart somewhat enlarged and softened. Osseous deposits in the semilunar valves of the aorta, and under the lining of left ventricle.

*Abdomen.*—Left kidney contained pus. The right iliac vein also had pus in it as high up as the vena cava. The crural vein was occupied by purulent matter, as well as the popliteal.

A large quantity of purulent matter was found under the fascia lata and in the intermuscular cellular tissue. The right hip and knee-joints both contained pus, and within the left shoulder-joint, and in the textures external to it, pus was likewise detected.

Were the "tubercular deposits," in the lungs, "secondary deposits?" We are inclined to think that the latter were mistakan for the former. Be that as it may, the case was clearly one of phlebitis and secondary deposits, from the operation. The question is, can such operations be justified? We are not sure of that. The risk is considerable, the utility far from assured. Mr. Cooper, rather an apologist than otherwise, for the operation, observes :—

"The case calls our attention to the question, under what circumstances is the attempt to cure varicose veins of the leg justifiable by any means attended with a risk of exciting this formidable disorder?

I should say, certainly not, if the varicose veins, and the ulcer connected with them, admit of relief from other plans, and are not producing severe annoyance, nor are generally interfering with a person's endeavour to gain a livelihood.

Then, if this kind of twisted suture be adopted, ought we to apply it at once in several places, or only in one, repeating it afterwards if another is necessary? The latter method is probably the safest. In particular, I advise you not to adopt the practice at all in unfavourable constitutions."

Perhaps Mr. Cooper's advice might be extended to not performing the operation at all in *any* constitution.

---

* Med. Gaz. Oct. 25, 1839.

